# Germ Cell Tumor of the Testis: Lethal Subtypes of a Curable Cancer

**DOI:** 10.3390/jcm13123436

**Published:** 2024-06-12

**Authors:** Jamaal C. Jackson, Darren Sanchez, Andrew C. Johns, Matthew T. Campbell, Ahmet M. Aydin, Neriman Gokden, Sanjay Maraboyina, Jason L. Muesse, John F. Ward, Louis L. Pisters, Niki M. Zacharias, Charles C. Guo, Shi-Ming Tu

**Affiliations:** 1 Department of Urology, The University of Texas MD Anderson Cancer Center, Houston, TX 77030, USA; jjackson10@mdanderson.org (J.C.J.); sanchezdarren@gmail.com (D.S.); jfward@mdanderson.org (J.F.W.); lpisters@mdanderson.org (L.L.P.); nmzacharias@mdanderson.org (N.M.Z.); 2 Department of Genitourinary Medical Oncology, The University of Texas MD Anderson Cancer Center, Houston, TX 77030, USA; acjohns@mdanderson.org (A.C.J.); mcampbell3@mdanderson.org (M.T.C.); 3 Division of Urology, University of Arkansas for Medical Sciences, Little Rock, AR 72205, USA; maydin@uams.edu; 4 Division of Pathology, University of Arkansas for Medical Sciences, Little Rock, AR 72205, USA; gokdenneriman@uams.edu; 5 Division of Radiation Oncology, University of Arkansas for Medical Sciences, Little Rock, AR 72205, USA; 6 Department of Surgery, University of Arkansas for Medical Sciences, Little Rock, AR 72205, USA; jmuesse@uams.edu; 7 Department of Pathology, The University of Texas MD Anderson Cancer Center, Houston, TX 77030, USA; ccguo@mdanderson.org; 8 Division of Hematology/Oncology, University of Arkansas for Medical Sciences, Little Rock, AR 72205, USA

**Keywords:** testicular cancer, sarcomatoid yolk sac tumor, epithelioid trophoblastic tumor, somatic transformation, intratumoral heterogeneity, drug resistance, therapy development

## Abstract

Germ cell tumor of the testis (GCT) is a curable cancer even when it is widely metastatic; however, outcomes can differ based on tumor histology. Chemo-resistance in certain phenotypes, such as teratoma and yolk sac tumor, contributes to poor clinical outcomes in some patients with GCT. Despite this resistance to S-YSTemic therapy, many of these tumor subtypes remain amenable to surgical resection and possible cure. In this study, we report on a series of seven patients highlighting two chemo-resistant subtypes of nonseminomatous germ cell tumor (NSGCT), sarcomatoid yolk sac tumor (S-YST), and epithelioid trophoblastic tumor (ETT) for which early resection rather than additional salvage chemotherapy or high-dose intense chemotherapy might provide a superior clinical outcome and enhance cure rate.

## 1. Introduction

Germ cell tumor of the testis (GCT) is a curable cancer even when it is widely metastatic. In a retrospective single institutional study, 63% (45/71) of those patients with clinical stage IIIC nonseminomatous germ cell tumor of the testis (NSGCT) were cured. However, 3% (14/429) of patients with clinical stage I-II NSGCTs died from an otherwise curable cancer [1]. It is evident that chemotherapy resistant GCT phenotypes, such as teratoma and yolk sac tumor, contributed to the poor clinical outcome of certain patients with GCTs [1,2]. Because these chemotherapy-resistant tumor subtypes may still be amenable to surgical resection with curative intent and there may be a window and threshold for surgery before the cancer becomes disseminated, it is imperative that we identify these potentially refractory tumor phenotypes to improve the cure rate of the remaining intractable GCT.

One type of chemo-resistant NSGCT is sarcomatoid yolk sac tumor (S-YST), which represents a likely cause of treatment failure in a small fraction of patients with GCT [3]. Damjanov first reported S-YST in 1984 [4]. S-YST stains positive for GPC3, AE1/AE3 (which tends to be negative in unclassified sarcomas), and SALL4, but negative for alpha fetoprotein (AFP) and MUC4. At the time of initial diagnosis, there is an absence of a teratomatous component in nearly half the cases of a primary GCT that harbors or evolves into S-YST. Furthermore, the spindle cell component of S-YST contains pluripotent cells with the capacity to form differentiated mesenchyme, including striated muscle [5].

Another chemo-resistant NSGCT subtype is epithelioid trophoblastic tumor (ETT). ETT arises from intermediate trophoblastic cells in the chorionic leaf. It stains positive for P63 and focally positive for human placental lactogen (hPL). Patients with ETT tend to have elevated beta-human chorionic gonadotropin (HCG) levels; however, their HCG level is generally lower (<2500 mIU/mL in 69% of cases) compared to patients with choriocarcinoma. Although ETT appears to display a benign behavior in most cases, it is known to be refractory to conventional chemotherapy [6]. Interestingly, Lewin and colleagues reported that all three patients with isolated pulmonary lesions and elevated HCG who underwent surgical resection were eventually free of disease [7]. Thus, resection of isolated metastasis is recommended when feasible. Conversely, delay in diagnosis and surgery may adversely impact survival [8,9].

In this study, we report on a small series highlighting two chemo-resistant subtypes of NSGCT, S-YST and ETT, for which early resection rather than additional salvage chemotherapy or high-dose intense chemotherapy might provide a superior clinical outcome and enhance cure rate. Our experience and available evidence suggest that early surgical extirpation of isolated tumors may be a more favorable option than adjuvant or salvage chemotherapy, which can cause significant morbidity and is usually ineffective in these tumors.

## 2. Case Reports

1. The patient was a 65-year-old Caucasian male diagnosed with a clinical stage IIX NSGCT. The primary tumor was diagnosed as seminoma, but the patient’s AFP was elevated. He subsequently received chemotherapy in the form of one cycle of bleomycin, etoposide, and cisplatin (BEP) and three cycles of cisplatin, etoposide, and ifosfamide (VIP). Six months following the diagnosis, the patient underwent retroperitoneal lymph node dissection (RPLND) and left radical nephrectomy—pathology showed teratoma. Three years later, pathology from a left neck lymph node (LN) dissection revealed teratoma. One month following the left nodal dissection, 8/11 LNs from a right thoracotomy showed teratoma.

**Figure 1:**
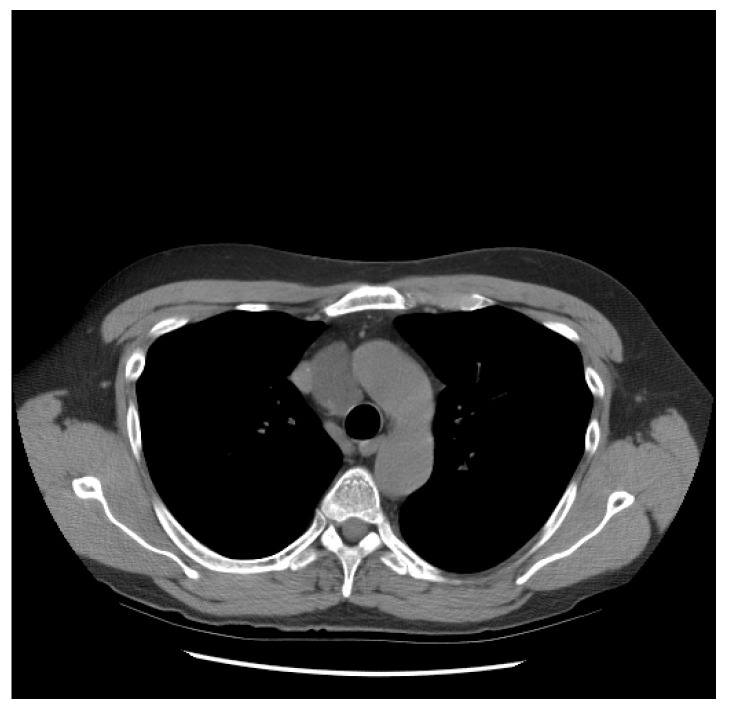
Patient S-YST 1 with a new upper mediastinal mass.

He did well until 21 years following the initial diagnosis, when he presented with a new upper mediastinal mass (Figure 1) and an AFP of 1048 ng/mL. He received paclitaxel, doxorubicin, and gemcitabine (he had a renal transplant due to renal failure after the prior nephrectomy and chemotherapy). Docetaxel replaced paclitaxel due to neuropathy; gemcitabine was removed due to low blood counts.

Twenty-two years after the diagnosis, the patient underwent a right thoracotomy and mediastinal LN dissection to remove residual disease. Pathology showed 90% teratoma, 10% ETT, and malignant transformation to adenocarcinoma. The patient did not receive any additional treatment after surgery and has remained without evidence of disease since his last surgery.

2. The patient was a 24-year-old Caucasian male with a stage IIIc NSGCT who presented with a six-month history of a progressive left testicular mass. He also reported a new neck mass, as well as left shoulder and arm pain. Family history was significant for Birt–Hogg–Dube syndrome. Initial serum tumor markers were notable for AFP 93,322 ng/mL and HCG 21 mIU/mL. The patient was taken to the operating room and underwent a left radical orchiectomy. Pathology revealed a 3.5 cm tumor composed of 5% embryonal carcinoma, 90% seminoma, and 5% yolk sac tumor. Post-orchiectomy staging computed tomography (CT) scan revealed a 22.5 cm retroperitoneal mass. The patient subsequently received four cycles of BEP chemotherapy. Tumor markers at this time had normalized with decrease in size of the retroperitoneal mass to 2.2 cm.

Three months after the initial surgery, the patient underwent a lymphadenectomy of the left neck which revealed only fibrosis and necrosis. Surveillance imaging remained stable until six months later, when staging work-up demonstrated the progression of disease in the retroperitoneum. Tumor markers remained within normal limits. The patient underwent RPLND 1 year following the initial diagnosis. Pathology showed a metastatic GCT comprised of 99% yolk sac tumor with sarcomatoid differentiation and 1% teratoma.

Routine surveillance imaging 10 months after the RPLND showed overall improvement of the prior lymphadenopathy, but three new left upper lobe lung nodules were noted (measuring up to 9 mm). A biopsy was performed which revealed metastatic cancer of testicular origin with yolk sac tumor versus immature teratoma pathology. He then proceeded to left upper lobectomy which showed a multifocal metastatic NSGCT with similar pathology to the previous lung biopsy.

One year following the upper lobectomy, a surveillance CT scan showed an enlarging 3 cm right periaortic LN suspicious for metastatic disease. The patient then underwent repeat RPLND two weeks later. Pathology was consistent with yolk sac tumor with sarcomatoid differentiation. He subsequently received consolidative radiation of the mediastinal operative bed. According to our records, the patient has remained free of disease to date.

**Figure 2:**
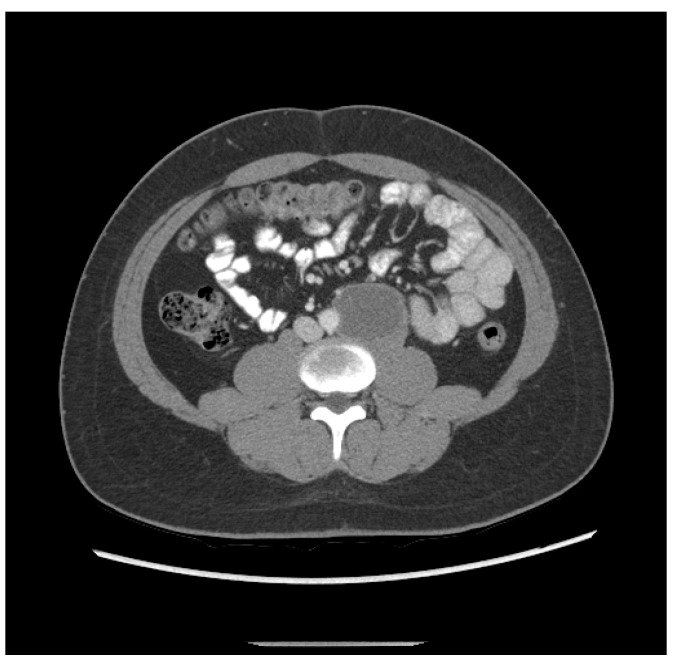
Patient ETT 3 with residual retroperitoneal lymphadenopathy after frontline chemotherapy.

3. The patient was a 20-year-old Hispanic male who complained of back pain for one week and presented to the local emergency department, which found a retroperitoneal mass measuring 6.3 cm and a left testicular mass. He then underwent a left radical orchiectomy. Pathology showed 80% epithelial trophoblastic tumor and 20% teratoma. He received three cycles of BEP. Staging CT after two cycles of BEP revealed shrinkage of the retroperitoneal mass to 5.2 cm (Figure 2). His initial tumor markers were unavailable; however, initial post-treatment HCG was 81.7 mIU/mL.

It was debated whether to treat him with salvage chemotherapy comprising of four cycles of cisplatin, ifosfamide, and paclitaxel (TIP) followed by RPLND to resect any residual tumor after normalization of HCG, or to proceed directly to RPLND, because his disease appeared to be chemo-resistant but still confined to the retroperitoneum. The patient decided to move forward with RPLND.

**Figure 3:**
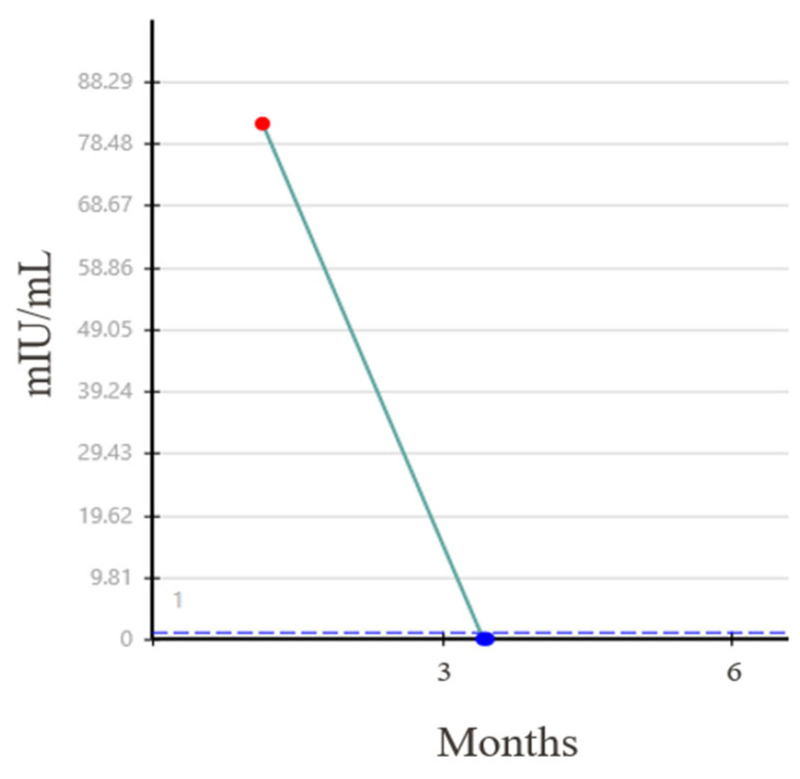
Normalization of beta HCG level after RPLND.

Three months following the initial diagnosis, the patient underwent an open RPLND. Three of 59 retroperitoneal lymph nodes (RPLN), measuring up to 3.0 cm, showed teratoma with minute foci of ETT. His HCG subsequently became undetectable (Figure 3) and he has remained without evidence of recurrent disease.

4. The patient was a 31-year-old Hispanic male with a stage IIIc NSGCT who presented to the emergency department with acute, severe back pain. He reported a three-year history of an enlarging left testicular mass at the time but deferred medical evaluation due to a lack of insurance. Initial CT imaging was notable for extensive lymphadenopathy and pulmonary and hepatic nodules; in addition to evidence of thrombosis in the left common iliac and left renal veins. Serum tumor markers were elevated with AFP 81,955 ng/mL, HCG 5489 mIU/mL, and lactate dehydrogenase (LDH) greater than 1000 U/L. The patient underwent a liver biopsy which revealed evidence of metastatic yolk sac tumor.

He was then started on four cycles of BEP chemotherapy. Post-chemotherapy serum tumor markers normalized except for AFP which decreased to 514.6 ng/mL. Despite variable radiographic responses, there was no appreciable change in the primary tumor, which remained at roughly 20 cm. A left radical orchiectomy was performed three months following the liver biopsy. Pathology showed viable, invasive teratoma. Given the extensive thrombosis, an inferior vena caval filter was placed by interventional radiology. He then underwent RPLND, left nephrectomy, excision of mesenteric mass, left hemicolectomy, and liver wedge resection five months after the initial diagnosis. Pathology revealed teratoma within 1/79 LN. Postoperatively, the patient was continued on rivaroxaban for five months.

The patient showed no evidence of disease until 37 months after the previous surgery when a staging work-up revealed new abdominal and thoracic lymphadenopathy. Fifteen months later, the patient presented with large-volume ascites and ultimately required paracentesis. Imaging at that time also revealed an intracardiac mass. Roughly one month after the paracentesis, he underwent abdominal LN biopsy and resection of intracardiac mass which revealed pathology consistent with S-YST. The patient continued to require frequent paracenteses. Two months following the last surgery, a staging CT demonstrated multiple intra-abdominal masses, including a 36 cm cystic mass causing inferolateral displacement of the colon. After assessment by Urology and the Sarcoma surgical service, the mass was deemed unresectable while maintaining the vital organs necessary for life. The patient ultimately decided to pursue available clinical trials versus hospice care and was lost to follow-up 60 months after the initial diagnosis.

5. The patient was a 26-year-old Hispanic male with a stage IIIA NSGCT who presented with a right testicular pain and a mass. Initial tumor markers were significant for HCG of 600 mIU/mL. Staging CT demonstrated disease within the retroperitoneum and lungs. He then underwent a right radical orchiectomy. Pathology showed 100% embryonal carcinoma. The patient subsequently received one cycle of BEP. He was unable to tolerate bleomycin and was transitioned to etoposide and cisplatin (EP) for an additional three cycles. Post-treatment HCG was undetectable.

The patient was subsequently lost to follow-up for six months. Tumor markers were found to be elevated on representation. Restaging PET/CT 31 months following the initial diagnosis revealed recurrent disease in the aortocaval LNs and the lungs. The patient then received salvage chemotherapy consisting of four cycles of TIP. This treatment was complicated by anemia, requiring a blood transfusion. Follow-up axial imaging demonstrated a residual 1.3 cm mass within the retroperitoneum and a new sub-centimeter right middle lung lobe nodule.

The patient then sought a second opinion at MD Anderson 36 months following the initial diagnosis. Tumor markers were within normal limits at this time; however, repeat axial imaging showed an increase in the size of the left upper lobe pulmonary metastasis with stability of the additional pulmonary and retroperitoneal lesions. One month after presenting to MD Anderson, his HCG had risen to 30.4 mIU/mL. The patient was started on two cycles of cisplatin, vincristine, methotrexate, and bleomycin (POMB) chemotherapy with radiographic evidence of response in all metastatic foci and normalization of HCG.

The patient then underwent left thoracotomy with left upper lobe segmentectomy and mediastinal lymphadenectomy to address persistent disease. Pathology was positive for metastatic embryonal carcinoma. HCG was found to be 25.8 mIU/mL one month following surgery. He was then given four cycles of ATP chemotherapy without radiographic response despite normalization of tumor markers. The patient was taken for RPLND four months following the prior thoracotomy. Pathology showed 1/10 LNs positive for metastatic carcinoma and ETT (measuring 3.8 cm) with invasion into the aortic wall and extranodal extension. Postoperative tumor markers taken one month after the RPLND were significant, with an HCG of 158.1 mIU/mL.

One month later, the patient underwent right upper lobe wedge resection and right middle lobectomy, the latter of which showed a 5.3 cm focus of choriocarcinoma. Surveillance PET/CT scan three months following the wedge resection revealed evidence of progressive metastatic disease with new pulmonary and liver lesions. In the setting of negative tumor markers, this raised a question for possible teratoma. The patient then received one cycle of TIP for stem cell mobilization, followed by two regimens of high-dose chemotherapy, with stem cell support. The first consisted of gemcitabine, docetaxel, melphalan, carboplatin; the second consisted of ifosfamide, carboplatin, and etoposide (ICE), respectively.

Despite these therapies, the patient succumbed to his disease 61 months following his initial surgery.

6. The patient was a 37-year-old Caucasian male diagnosed with a clinical stage IIIA NSGCT. Past medical history was significant for right cryptorchidism, for which he underwent orchiopexy at ages 8 and 19. A right radical orchiectomy was performed. Pathology revealed 99% embryonal carcinoma and 1% teratoma. Postoperative HCG was 207 mIU/mL. On staging CT, right inguinal (measuring 2.5 cm) and femoral (2.0 cm) lymphadenopathy was noted. The patient then received three cycles of BEP before undergoing consolidative right inguinal and femoral lymphadenectomy seven months after the diagnosis. Pathology ultimately showed teratoma. Postoperative tumor markers were undetectable, and the patient remained disease-free until 14 years later, when he was found to have recurrent disease within a right inguinal LN. HCG at the time was 115 mIU/mL. He then underwent right inguinal lymph node dissection (ILND). Pathology showed embryonal carcinoma and choriocarcinoma. Repeat HCG one month later was 224 mIU/mL. He then received salvage chemotherapy in the form of TIP (three cycles).

Three months after the recurrence, repeat staging imaging was obtained via PET/CT which demonstrated a metabolically active left inguinal LN, in addition to multiple inactive lung nodules. One month later, he underwent left ILND. Pathology showed no viable disease. In the interim, HCG had incrementally increased from 15.5 mIU/mL to 183.8 mIU/mL within a two-month span. The patient was then started on doxorubicin, paclitaxel, and cisplatin chemotherapy. During the treatment course, paclitaxel was replaced by docetaxel due to neuropathy.

Interim staging imaging showed interval resolution of some pulmonary LNs with concurrent enlargement of others, raising suspicion of metastatic disease. Six months following the last ILND, he underwent wedge resection of the residual right lung lesions in which 5/7 lesions (measuring up to 2.8 cm) showed up to 60% viable tumor comprising ETT vs. choriocarcinoma.

**Figure 4:**
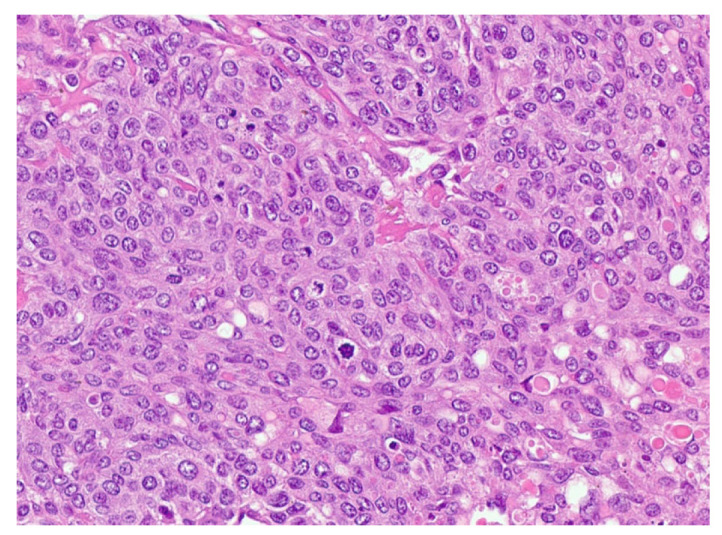
Epithelioid trophoblastic tumor shows relatively uniform intermediate trophoblast cells with eosinophilic hyaline-like material.

One month later, he returned for wedge resection of residual left lung lesions in which 11/13 lesions (measuring up to 5.1 cm) contained up to 60% viable tumor comprising ETT with somatic transformation to poorly differentiated carcinoma/squamous cell carcinoma (Figure 4).

**Figure 5:**
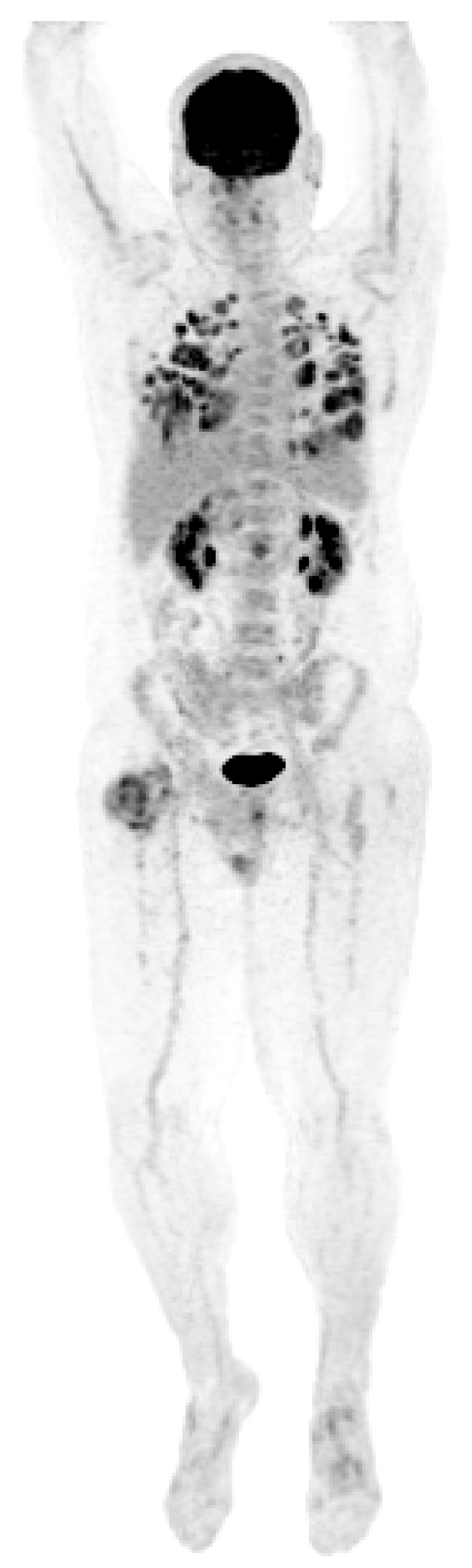
PET scan showing diffuse lung metastases and a right proximal femoral metastasis.

A new hypermetabolic lesion in the right intertrochanteric region was observed on PET/CT two months following the second wedge resection. A CT-guided biopsy of the lesion was consistent with metastatic GCT. The patient developed widely disseminated recurrent disease (Figure 5) and he ultimately expired one month later at 190 months following his initial diagnosis.

7. The patient was a 35-year-old African American male who presented with a three-month history of abdominal pains and was found to have a retroperitoneal mass on cross sectional imaging. Ultrasonography showed no testicular lesion. Upper and lower gastrointestinal endoscopic evaluation was unrevealing. Two biopsies of the retroperitoneal mass were nondiagnostic. Review of his pathology showed tumor positivity for i(12p), suggestive of somatic transformation of the GCT to adenocarcinoma.

**Figure 6:**
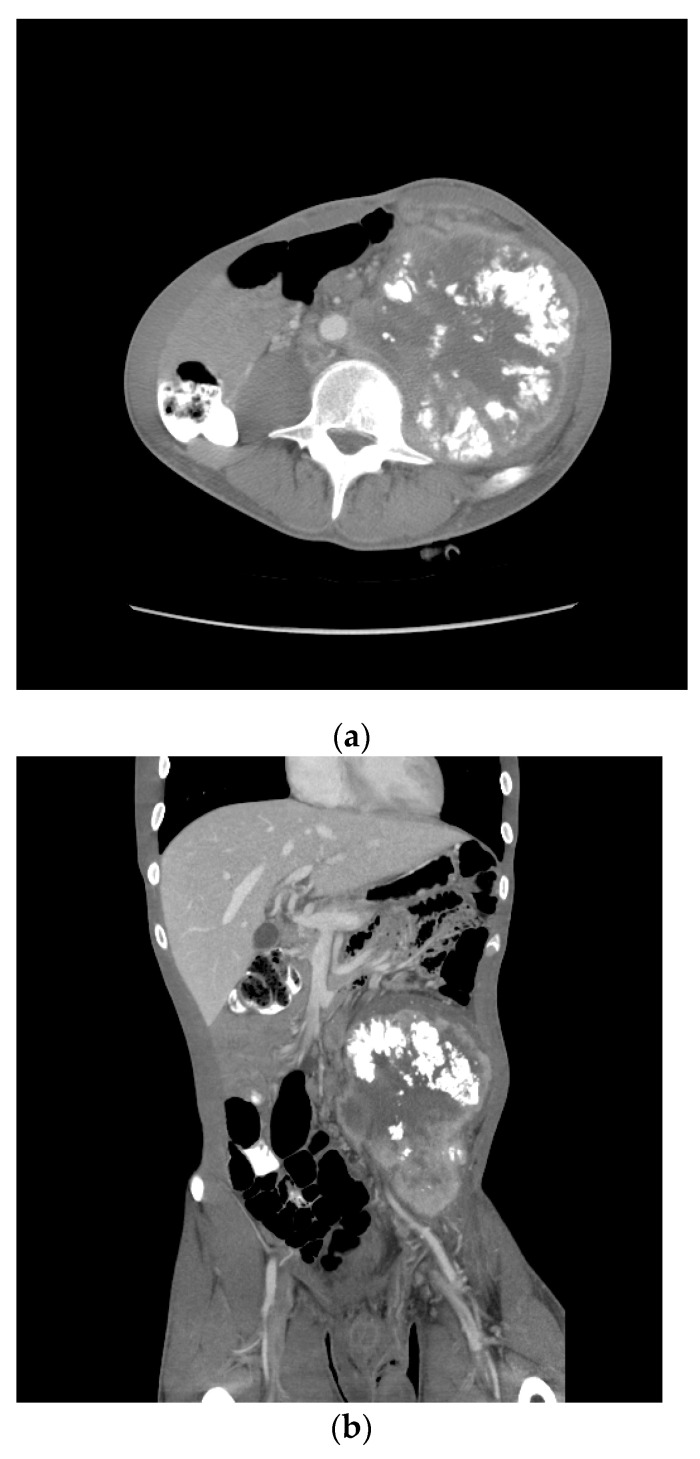
Patient S-YST 2 with bulky calcified lymphadenopathy ((**a**) transverse and (**b**) coronal sections).

One month later, he received two cycles of BEP without benefit. Four months after the initial therapy, he received capecitabine and oxaliplatin (XELOX), again without benefit. By then, the retroperitoneal mass had increased in size from 12 to 18 cm over the past year. Eight months following the initial diagnosis, his AFP was 205.3 ng/mL, HCG 4.4 mIU/mL, LDH 385 U/L. A CT scan showed a bulky left psoas mass, multicompartmental lymphadenopathy involving bilateral retroperitoneum, left common iliac, left pelvic, left gonadal, and inferior mesenteric chains without overt solid organ metastasis (Figure 6).

**Figure 7:**
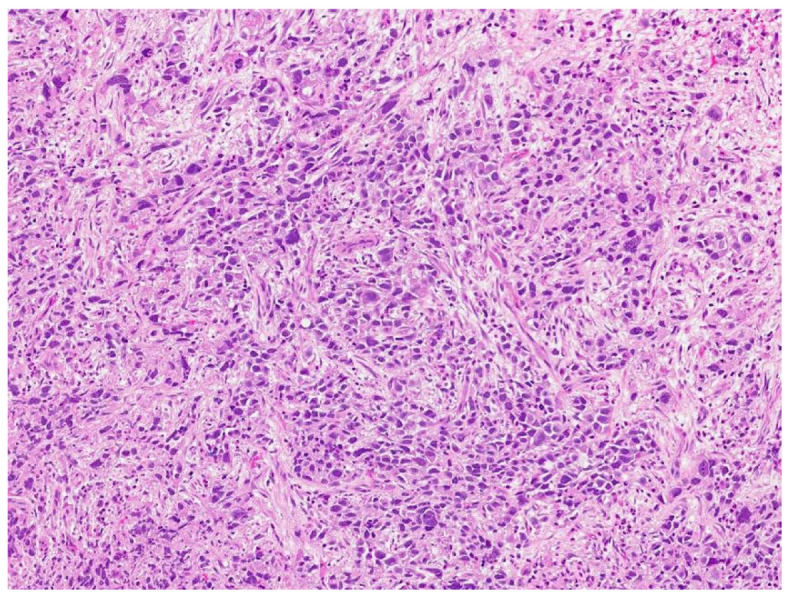
Sarcomatoid yolk sac tumor with pleomorphic epithelioid and spindle cells, hematoxylin, and eosin stain.

**Figure 8:**
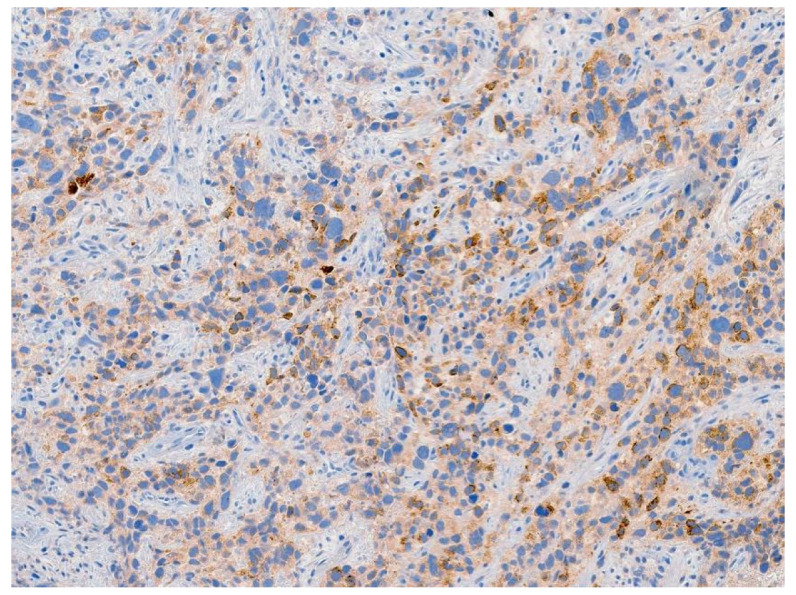
Tumor stained positive for glypican-3, a specific immunohistochemical marker for yolk sac tumor.

Review of pathology indicated S-YST (Figure 7): SALL4, focally positive; GPC3, weakly and focally positive (Figure 8). CD117 and OCT3/4 were negative in tumor cells, i(12p) was positive.

Two cycles of doxorubicin, paclitaxel, and cisplatin (ATP) were administered that same month. The following month, he received palliative radiation to L3 and embolization of the symptomatic tumor. The patient expired 12 months after the initial diagnosis.

## 3. Discussion

A major goal in the management of GCTs is to maintain a high cure rate with less therapeutic burden, including the associated treatment toxicity and impact on patient quality of life. When it concerns refractory GCTs, especially after salvage TIP and/or high-dose chemotherapy with stem cell transplant, current NCCN and ESMA guidelines offer a similar recommendation: consider clinical trials or palliative/hospice care [10,11]. Unfortunately, neither guideline elaborates about the types and nature of refractory GCTs, such as S-YSTs and ETTs, due to their rarity and our lack of understanding and awareness of such lethal entities. This highlights the importance of tailoring therapeutic options to suit the disease process. One way to accomplish this is to identify those cases of potentially lethal GCTs that may prove unresponsive to current treatment modalities and require alternative therapeutic strategies. Accomplishing this requires further investigation to gain a deeper understanding of the basic biology of GCTs; their intratumoral and intertumoral heterogeneity, genetic and epigenetic origins; and factors that contribute to chemo-sensitivity, or lack thereof.

It has been shown that refractory S-YSTs and ETTs can have a relatively indolent course and may still be organ confined for a prolonged period. This observation suggests that certain patients with late relapses of GCTs are still potentially salvageable by surgery, as seen in cases 1–3 (Table 1) [12]. The biology of GCTs is such that certain indolent tumors in the testis (stage I) and in the retroperitoneum (stage II) may still be curable by surgery even though they are intrinsically refractory to S-YSTemic treatments such as chemotherapy. Therefore, it is imperative to incorporate the unique biological characteristics of each tumor into clinical decision-making to achieve optimal therapeutic outcomes.

It may not be coincidental that both S-YSTs and ETTs tend to coexist with teratomas and somatically transformed tumors. Each of these tumor phenotypes are chemo-resistant, which contributes to their increased lethality. Furthermore, certain NSGCTs are predisposed to undergo somatic transformation and predestined to worse clinical outcomes. Despite the generally poorer prognosis in patients with these variants, outcomes among this cohort are not uniform. Patients found to have evidence of somatic transformation identified in the primary tumor following radical orchiectomy fared better when compared to those who first displayed this transformation in metastatic lesions [1]. The nature of S-YSTs and ETTs in mixed tumors and similarities to mature teratoma raises the question of origin of tumor heterogeneity in this phenomenon—that is, whether a differentiated malignant teratoma dedifferentiates when it undergoes somatic transformation or whether an undifferentiated malignant progenitor germ cell undergoes somatic differentiation to form a teratoma as well as other GCT and non-GCT lineages [13].

In the 1940s, about 90% of patients with metastatic GCTs died within one year of diagnosis [14]. The cure rate was roughly 60% in the 1970s with the use of cisplatin, vinblastine, and bleomycin (PVB) [15]. Contemporary data shows over 90% of patients with the same diagnosis are cured [1]. These improved outcomes are largely due to the refinement of platinum-based chemotherapy regimens. However, it is also evident that the improved cure rate of GCTs is also a reflection of improved multimodal treatment algorithms. Substituting etoposide for vinblastine in chemotherapeutic regimens (i.e., BEP versus PVB) resulted in statistically improved response rates but similar rates of complete response and two-year survival [16]. Additionally, VIP (using ifosfamide instead of etoposide) [17,18] and a dose-dense regimen (adding paclitaxel) [19] were shown to be equal to BEP.

Thus, these improved clinical outcomes, including increased cure rates, can be largely attributed to improved cross-sectional imaging, surgical and perioperative care, staging with incorporation of serum markers, and application of multimodal approaches rather than development of novel drug regimens [20]. For example, up to 80% of NSGCT are mixed tumors. In its embryonal component, a mixed NSGCT may have the same genetic makeup due to a common clonal origin (e.g., presence of i(12p)) which is exquisitely chemo-sensitive, while its teratoma component remains chemo-resistant [21]. The approach to a tumor such as this one may involve eradication of the embryonal component with S-YSTemic chemotherapy, followed by surgical resection to address the residual teratoma component post-chemotherapy. Given the intra- and intertumoral heterogeneity, a multimodal therapeutic approach may be more likely to achieve durable response rates than treatments targeting specific genetic aberrations [i.e., i(12p)]. Previous data has also shown that “precision medicine” (e.g., targeting c-kit, CD-30) is more likely to provide only transient responses and modest control of GCTs [22,23].

A number of mutations have been observed within GCTs which may be ideal for targeted therapies including mutations in mismatch repair genes, KRAS, and most commonly KIT [24,25,26,27,28,29,30]. Furthermore, 73% of seminoma and 64% of nonseminoma express PD-L1 [29,30]. However, a phase II multi-institutional trial showed that most patients with refractory PD-L1+ TGCTs did not respond to anti-PD1/L1 therapy [30], while targeted therapy (against KIT, CD30) has so far failed to provide any tangible clinical benefits [22,23,31]. Antibody–drug conjugate (ADC) targeting TROP-2 against ETTs and glypican-3 (GPC3) against S-YSTs deserves further investigation. In one study, all yolk sac tumor (24/24) and choriocarcinoma (7/7) components were immunoreactive for GPC3 and 38% of immature teratomas and 8% of embryonal carcinomas expressed GPC3, while none of the benign testicular tissue, intratubular germ cell neoplasia, seminoma (0/42), or mature teratomas (0/20) showed expression [32]. These novel therapies remain in development but may present another viable treatment option in this select patient population.

## 4. Conclusions

In conclusion, a multimodal approach, including timely surgical intervention, is likely essential in achieving improved cure rates in patients with potentially lethal subtypes of NSGCTs, such as S-YSTs and ETTs. Further study into the etiological origin and biological nature of these subtypes are needed to further refine the global treatment paradigm in this cohort of patients.

## Figures and Tables

**Table 1:** Consecutive cases of ETTs and S-YSTs between July 1990 and January 2019 at a single institution: clinical and pathological outcomes.

Patient NSGCT Type	Status	Pathology of Primary	Stage at Time of Diagnosis	S-YSTemic Chemo	Date: Pathology after Chemo
1 ETT	Cured	100% S (elevated AFP)	IIX	BEP x1, VIP x3, paclitaxel (docetaxel), doxorubicin, gemcitabine,	6/2012: 90% T, 10% ETT + adenocarcinoma
2 S-YST	Cured	5% E, 90% S, 5% Y	IIIC	BEP x4	5/2018, 4/2019, 4/2020: 99% S-YST + 1% T
3 ETT	Cured	80% ETT, 20% T	IIC	BEP x3	1/2018: T w/minute ETT
4 S-YST	Died	NA Y in met	IIIC	BEP x4	9/2016: T 1/2021: S-YST
5 ETT	Died	100% E	IIIA	BEP x1, EP x3, TIP x4, POMB x2, ATP x4, TIP x1/HDC + SCT	7/2019: Embryonal 11/2019: ETT 1/2020: Choriocarcinoma
6. ETT	Died	99% E, 1% T	IIIA	BEP x3, TIP x3, ATP	9/2018: E, C 7/2019, 8/2019: 60% ETT + squamous cell carcinoma
7 S-YST	Died	NA i(12p)+ in met	IIC	BEP x2, XELOX, ATP x2	8/2019: S-YST (Unresectable)

ETT: epithelial trophoblastic tumor; S-YST, sarcomatoid yolk sac tumor; S, seminoma, E, embryonal carcinoma; T, teratoma; Y, yolk sac tumor; C, choriocarcinoma; BEP, bleomycin, etoposide, cisplatin; VIP, etoposide, ifosphamide, cisplatin; POMB, methotrexate, bleomycin, vincristine, cisplatin; ATP, doxorubicin, paclitaxel, cisplatin; HDC + SCT, high-dose chemo + stem cell transplant; XELOX, capecitabine, oxaliplatin.

## Data Availability

The data in this study were generated by the authors and are available upon request from the corresponding author.

## Abbreviations

AFP	Alpha fetoprotein
BEP	Bleomycin, etoposide, cisplatin
CT	Computed tomography
EP	Etoposide and cisplatin
ETT	Epithelioid trophoblastic tumor
GCT	Germ cell tumor
HCG	Beta-human chorionic gonadotropin
ILND	Inguinal lymph node dissection
LDH	Lactate dehydrogenase
LN	Lymph node
NSGCT	Nonseminomatous germ cell tumor
POMB	Cisplatin, vincristine, methotrexate, and bleomycin
RPLN	Retroperitoneal lymph node
RPLND	Retroperitoneal lymph node dissection
S-YST	Sarcomatoid yolk sac tumor
TIP	Paclitaxel, ifosfamide, cisplatin
VIP	Cisplatin, etoposide, ifosfamide

## References

[1] Tu S.M., Bilen M.A., Hess K.R., Broaddus R.R., Kopetz S., Wei C., Pagliaro L.C., Karam J.A., Ward J.F., Wood C.G. (2016). Intratumoral heterogeneity: Role of differentiation in a potentially lethal phenotype of testicular cancer. Cancer.

[2] Funt S.A., Patil S., Feldman D.R., Motzer R.J., Bajorin D.F., Sheinfeld J., Tickoo S.K., Reuter V.E., Bosl G.J. (2019). Impact of Teratoma on the Cumulative Incidence of Disease-Related Death in Patients with Advanced Germ Cell Tumors. J. Clin. Oncol..

[3] Howitt B.E., Magers M.J., Rice K.R., Cole C.D., Ulbright T.M. (2015). Many postchemotherapy sarcomatous tumors in patients with testicular germ cell tumors are sarcomatoid yolk sac tumors: A study of 33 cases. Am. J. Surg. Pathol..

[4] Damjanov I., Amenta P.S., Zarghami F. (1984). Transformation of an AFP-positive yolk sac carcinoma into an AFP-negative neoplasm. Evidence for in vivo cloning of the human parietal yolk sac carcinoma. Cancer.

[5] Michael H., Ulbright T.M., Brodhecker C.A. (1989). The Pluripotential Nature of the Mesenchyme-Like Component of Yolk-Sac Tumor. Arch. Pathol. Lab. Med..

[6] Keser S.H., Kokten S.C., Cakir C., Sensu S., Buyukbayrak E.E., Karadayi N. (2015). Epithelioid trophoblastic tumor. Taiwan J. Obstet. Gynecol..

[7] Lewin S.N., Aghajanian C., Moreira A.L., Soslow R.A. (2009). Extrauterine epithelioid trophoblastic tumors presenting as primary lung carcinomas: Morphologic and immunohistochemical features to resolve a diagnostic dilemma. Am. J. Surg. Pathol..

[8] Jordan S., Randall L.M., Karamurzin Y., Ward P., Lin F., Brewster W., Monk B.J. (2011). Differentiating squamous cell carcinoma of the cervix and epithelioid trophoblastic tumor. Int. J. Gynecol. Cancer.

[9] Shet T., Parage M., Maheshwari A., Nair R., Gupta S., Tongaonkar H., Chinoy R. (2008). Epithelioid trophoblastic tumor of uterus presenting as an ovarian mass: A diagnostic and therapeutic dilemma. Indian J. Pathol. Microbiol..

[10] Gilligan T., Lin D.W., Aggarwal R., Chism D., Cost N., Derweesh I.H., Emamekhoo H., Feldman D.R., Geynisman D.M., Hancock S.L. (2019). Testicular Cancer, Version 2.2020, NCCN Clinical Practice Guidelines in Oncology. J. Natl. Compr. Cancer Netw..

[11] Oldenburg J., Berney D., Bokemeyer C., Climent M., Daugaard G., Gietema J., De Giorgi U., Haugnes H., Huddart R., Leão R. (2022). Testicular seminoma and non-seminoma: ESMO-EURACAN Clinical Practice Guideline for diagnosis, treatment and follow-up. Ann. Oncol..

[12] Moore J.A., Slack R.S., Lehner M.J., Campbell M.T., Shah A.Y., Zhang M., Guo C.C., Ward J.F., Karam J.A., Wood C.G. (2022). Very Late Recurrence in Germ Cell Tumor of the Testis: Lessons and Implications. Cancers.

[13] Tu S.M., Zhang M., Wood C.G., Pisters L.L. (2021). Stem Cell Theory of Cancer: Origin of Tumor Heterogeneity and Plasticity. Cancers.

[14] Friedman N.B., Moore R.A. (1947). Tumors of the Testis—A Report on 922 Cases. J. Urol..

[15] Einhorn L.H., Donohue J. (1977). Cis-diamminedichloroplatinum, vinblastine, and bleomycin combination chemotherapy in disseminated testicular cancer. Ann. Intern. Med..

[16] Williams S.D., Birch R., Einhorn L.H., Irwin L., Greco F.A., Loehrer P.J. (1987). Treatment of disseminated germ-cell tumors with cisplatin, bleomycin, and either vinblastine or etoposide. N. Engl. J. Med..

[17] de Wit R., Stoter G., Sleijfer D., Neijt J., Huinink W.T.B., de Prijck L., Collette L., Sylvester R. (1998). Four cycles of BEP vs four cycles of VIP in patients with intermediate-prognosis metastatic testicular non-seminoma: A randomized study of the EORTC Genitourinary Tract Cancer Cooperative Group. Br. J. Cancer.

[18] Nichols C.R., Catalano P.J., Crawford E.D., Vogelzang N.J., Einhorn L.H., Loehrer P.J. (1998). Randomized comparison of cisplatin and etoposide and either bleomycin or ifosfamide in treatment of advanced disseminated germ cell tumors: An Eastern Cooperative Oncology Group, Southwest Oncology Group, and Cancer and Leukemia Group B Study. J. Clin. Oncol..

[19] Fizazi K., Pagliaro L., Laplanche A., Fléchon A., Mardiak J., Geoffrois L., Kerbrat P., Chevreau C., Delva R., Rolland F. (2014). Personalised chemotherapy based on tumour marker decline in poor prognosis germ-cell tumours (GETUG 13): A phase 3, multicentre, randomised trial. Lancet Oncol..

[20] Tu S.M., Pisters L.L. (2021). Curing Cancer: Lessons from a Prototype. Cancers.

[21] Kernek K.M., Ulbright T.M., Zhang S., Billings S.D., Cummings O.W., Henley J.D., Michael H., Brunelli M., Martignoni G., Foster R.S. (2003). Identical allelic losses in mature teratoma and other histologic components of malignant mixed germ cell tumors of the testis. Am. J. Pathol..

[22] Einhorn L.H., Brames M.J., Heinrich M.C., Corless C.L., Madani A. (2006). Phase II study of imatinib mesylate in chemotherapy refractory germ cell tumors expressing KIT. Am. J. Clin. Oncol..

[23] Necchi A., Anichini A., Raggi D., Giannatempo P., Magazzù D., Nicolai N., Colecchia M., Paolini B., Coradeschi E., Tassi E. (2016). Brentuximab Vedotin in CD30-Expressing Germ Cell Tumors After Chemotherapy Failure. Clin. Genitourin. Cancer.

[24] Tu S.M. (2010). Origin of cancers. Clinical perspectives and implications of a stem-cell theory of cancer. Cancer Treat. Res..

[25] Bilen M.A., Hess K.R., Campbell M.T., Wang J., Broaddus R.R., Karam J.A., Ward J.F., Wood C.G., Choi S.L., Rao P. (2016). Intratumoral heterogeneity and chemoresistance in nonseminomatous germ cell tumor of the testis. Oncotarget.

[26] Tu S.-M. (2019). The Story of Hydra: Portrait of Cancer as a Stem-Cell Disease. Cancer Etiology, Diagnosis and Treatments.

[27] Velasco A., Riquelme E., Schultz M., Wistuba I.I., Villarroel L., Pizarro J., Berlin A., Ittmann M., Koh M.S., Leach F.S. (2004). Mismatch repair gene expression and genetic instability in testicular germ cell tumor. Cancer Biol. Ther..

[28] McIntyre A., Summersgill B., Grygalewicz B., Gillis A.J., Stoop J., van Gurp R.J., Dennis N., Fisher C., Huddart R., Cooper C. (2005). Amplification and overexpression of the KIT gene is associated with progression in the seminoma subtype of testicular germ cell tumors of adolescents and adults. Cancer Res..

[29] Fankhauser C.D., Curioni-Fontecedro A., Allmann V., Beyer J., Tischler V., Sulser T., Moch H., Bode P.K. (2015). Frequent PD-L1 expression in testicular germ cell tumors. Br. J. Cancer.

[30] Cierna Z., Mego M., Miskovska V., Machalekova K., Chovanec M., Svetlovska D., Hainova K., Rejlekova K., Macak D., Spanik S. (2016). Prognostic value of programmed-death-1 receptor (PD-1) and its ligand 1 (PD-L1) in testicular germ cell tumors. Ann. Oncol..

[31] Adra N., Einhorn L.H., Althouse S.K., Ammakkanavar N.R., Musapatika D., Albany C., Vaughn D., Hanna N.H. (2018). Phase II trial of pembrolizumab in patients with platinum refractory germ-cell tumors: A Hoosier Cancer Research Network Study GU14-206. Ann. Oncol..

[32] Zynger D.L., Dimov N.D., Luan C., Teh B.T., Yang X.J. (2006). Glypican 3: A novel marker in testicular germ cell tumors. Am. J. Surg. Pathol..

